# Current applications of tumor local ablation (TLA) combined with immune checkpoint inhibitors in breast cancer treatment

**DOI:** 10.20517/cdr.2024.77

**Published:** 2024-09-13

**Authors:** Lingpeng Tang, Dandan Wang, Ting Hu, Xiaoying Lin, Songsong Wu

**Affiliations:** ^1^Department of Ultrasonography, Shengli Clinical Medical College of Fujian Medical University, Fujian Provincial Hospital, Fuzhou University Affiliated Provincial Hospital, Fuzhou 350001, Fujian, China.; ^2^Department of Ultrasound, The Second Affiliated Hospital of Fujian Medical University, Quanzhou 362000, Fujian, China.; ^#^Authors contributed equally.

**Keywords:** Tumor microenvironment, antitumor immune response, ablation, immune checkpoint inhibitors

## Abstract

Breast cancer is one of the most common cancers in women globally, posing significant challenges to treatment because of the diverse and complex pathological and molecular subtypes. The emergence of immune checkpoint inhibitors (ICIs) has revolutionized the treatment of breast cancer, particularly for triple-negative breast cancer (TNBC), significantly improving patient outcomes. However, the overall tumor response rate remains suboptimal due to drug resistance to ICIs. This resistance is primarily due to the immune-suppressive tumor microenvironment (TME), tumor cells’ ability to evade immune surveillance, and other complex immune regulatory mechanisms. To address these challenges, clinical researchers are actively exploring combinatorial therapeutic strategies with ICIs. Tumor local ablation (TLA) technology is anticipated to overcome resistance to ICIs and enhance therapeutic efficacy by ablating tumor tissue, releasing tumor antigens, remodeling the TME, and stimulating local and systemic immune responses. Combination therapy with TLA and ICIs has demonstrated promising results in preclinical breast cancer studies, underscoring the feasibility and importance of addressing drug resistance mechanisms in breast cancer. This provides novel strategies for breast cancer treatment and is expected to drive further advancements in the field.

## INTRODUCTION

Breast cancer ranks among the three most common malignancies globally, along with lung and colon cancer^[1-3]^. According to the Cancer Yearbook 2022, approximately 2.3 million new cases of breast cancer were reported worldwide, and the annual death toll from this disease exceeds 660,000^[4]^. Although surgery effectively removes primary tumors, it is often ineffective and invasive for tiny metastatic lesions. Surgery remains the primary treatment option for early-stage breast cancer^[5-7]^. Chemotherapy targets rapidly dividing cells systemically, but its non-selectivity can damage normal tissues, leading to severe systemic adverse effects such as myelosuppression, nausea, vomiting, and hair loss. Additionally, tumor cell resistance limits the long-term efficacy of chemotherapy^[8,9]^. Radiotherapy primarily targets tumor cells through localized irradiation; however, it may also damage surrounding healthy tissues, leading to long-term complications such as radiation fibrosis and cardiovascular damage. While radiotherapy is effective in controlling local tumor growth, it can also damage surrounding healthy tissues and cause additional complications^[10,11]^. Consequently, researchers have been seeking new, highly targeted treatments with fewer side effects.

In recent years, tumor immunotherapy, particularly immune checkpoint inhibitors (ICIs), has garnered significant attention due to their unique mechanisms of action^[12-18]^. ICIs activate T-cell-mediated antitumor immune responses by inhibiting immune checkpoint molecules such as programmed death receptor-1 (PD-1)/programmed death ligand-1 (PD-L1) and cytotoxic T-lymphocyte-associated protein 4 (CTLA-4). However, breast cancer is often considered a “cold tumor”, characterized by low immune cell infiltration and generally poor response to immunotherapy^[19,20]^. Nevertheless, triple-negative breast cancer (TNBC) presents potential opportunities for the application of ICIs due to its higher levels of infiltrating lymphocytes, increased tumor mutational burden (TMB), and elevated PD-L1 expression^[21-23]^.

However, a significant challenge facing ICIs in breast cancer treatment is the development of drug resistance. This resistance can be classified as primary or acquired, with mechanisms including: immunosuppression of the tumor microenvironment (TME) [e.g., recruitment of regulatory T cells (Tregs) and myeloid-derived suppressor cells (MDSCs), secretion of suppressor cytokines like transforming growth factor β (TGF-β) and interleukin 10 (IL-10)]^[24]^; loss of tumor antigenicity, leading to immune evasion; dynamic changes in immune checkpoint molecule expression (e.g., upregulation of other checkpoints like LAG-3, TIM-3); alterations in tumor cell signaling pathways (e.g., activation of the PI3K-AKT-mTOR pathway); tumor heterogeneity; and changes in the intestinal flora. These resistance mechanisms render ICIs less effective as monotherapy in breast cancer^[25-27]^. Consequently, ICIs must be combined with other therapies to maximize their antitumor efficacy and improve survival rates in breast cancer patients.

Current studies indicate that tumor local ablation (TLA) demonstrates significant efficacy and safety in the treatment of breast cancer^[28,29]^. It allows for localized treatment without the need for a surgical incision by using image-guided ablation electrodes to act directly on the tumor tissue. Compared to traditional surgery, TLA has the advantages of less trauma, faster recovery, and no scarring. However, ablation technology can be applied not only for the local treatment of tumors but also to stimulate antitumor immune responses in the body, providing systemic therapeutic potential for tumor treatment. It has been shown that TLA is able to destroy tumor cells through hyperthermia or rapid cooling, leading to cell death and the release of tumor antigens^[30,31]^. These antigens are captured by the dendritic cells (DCs) of the immune system and presented to T cells, activating the immune response. T cells then recognize and attack tumor cells containing the same antigen, enabling a systemic attack on the tumor. This process also promotes the formation of immune memory and enhances the body’s long-term defense against tumors. It is now being actively explored to enhance the efficacy of ICIs^[32,33]^.

**Figure 1:**
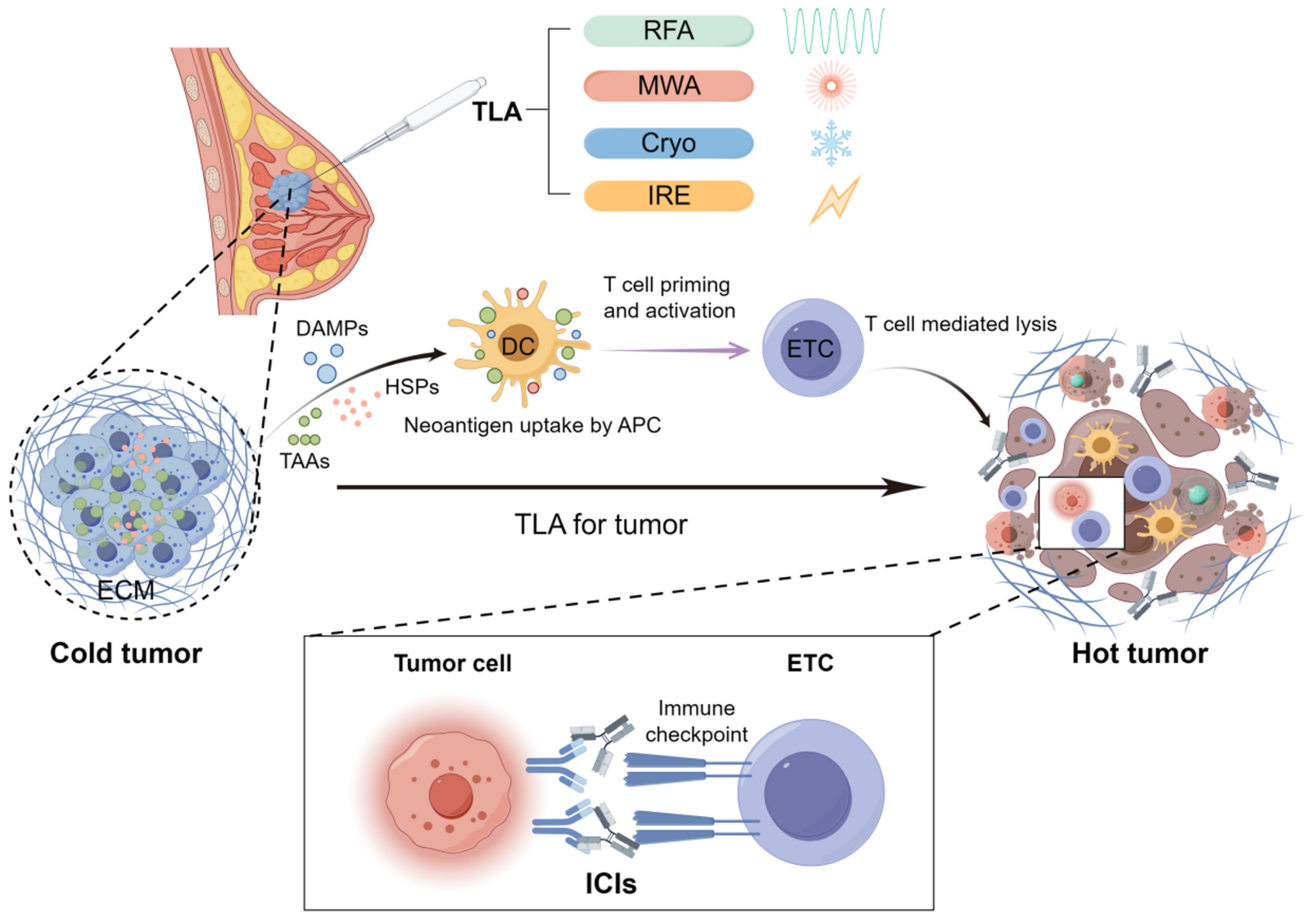
Schematic diagram of TLA combined with ICIs for the treatment of breast cancer. TLA: Tumor local ablation; RFA: radiofrequency ablation; MWA: microwave ablation; Cryo: cryoablation; IRE: irreversible electroporation; ECM: extracellular matrix; DCs: dendritic cells; ETC: effector T cells; ICIs: immune checkpoint inhibitors. Created with Figdraw 2.0 (ID: UYSTSb8a6b).

The combination of TLA and ICIs for the treatment of breast cancer represents a therapeutic approach that integrates ablation techniques and immunotherapy. This strategy aims to destroy tumor tissue through ablation technology, leading to damage and necrosis, release of antigens, and enhanced recognition and attack by the immune system. Conversely, ICIs modulate the function of the immune system and inhibit the tumor’s immune escape mechanisms, thereby enhancing the effectiveness of the combination therapy [Figure 1]. In this article, we first outline the efficacy of ICIs and TLAs and their limitations in breast cancer treatment. We then review the current status of combining ICIs and TLAs in breast cancer therapy and discuss future prospects.

## MECHANISMS OF DRUG RESISTANCE IN BREAST CANCER

Breast cancer resistance to treatment is a multifactorial, multilevel, and complex process. According to the time of its emergence and characteristics, it can be categorized into primary resistance and acquired resistance^[34]^. Primary resistance refers to resistance that exists before or at the beginning of treatment; for example, some breast cancer cells are born with *ESR1* gene mutations^[35]^. Conversely, acquired resistance develops gradually over the course of treatment; for example, acquired anti-human epidermal growth factor receptor 2 (HER2) resistance remains a major obstacle to the treatment of HER2-positive breast cancer^[36]^. Understanding these mechanisms of resistance is crucial for developing effective therapeutic strategies.

### Common mechanisms of resistance

Resistance to breast cancer involves various therapeutic areas, including endocrine therapy, chemotherapy, targeted therapy, and radiotherapy. Endocrine therapy resistance primarily stems from alterations in the estrogen receptor (ER) signaling pathway^[37]^. In addition, activation of growth factor receptor signaling pathways, such as the overexpression of HER2 and epidermal growth factor receptor (EGFR), and dysregulation of cell cycle regulatory mechanisms, are also important factors^[38-40]^. Chemotherapy resistance is commonly associated with multiple mechanisms. Among them, overexpression of drug efflux pumps is particularly important, notably the upregulation of P-glycoprotein^[41]^. In addition, enhanced DNA repair mechanisms and dysregulation of apoptotic pathways also contribute significantly to chemotherapy resistance^[42]^. The development of resistance to targeted therapy is often associated with mutations in the target, for example, mutations in the *HER2* gene^[38]^. Radiotherapy resistance, on the other hand, may arise due to the enhanced DNA repair capacity of tumor cells, the presence of cancer stem cells, and alterations in the TME^[43]^.

### Mechanisms of resistance to ICIs

As ICIs are increasingly applied in breast cancer treatment, their resistance mechanisms are gradually being revealed. Resistance to ICIs can be categorized as primary or acquired. Primary resistance is often closely related to the immunosuppressive state of the TME. Various immunosuppressive cells, including Tregs, MDSCs, and tumor-associated macrophages (TAMs), are present in the breast cancer microenvironment. These cells secrete inhibitory cytokines, such as TGF-β and IL-10, which collectively suppress the function of effector T cells^[44]^. Additionally, a lack of tumor antigenicity is a significant cause of primary drug resistance. Some breast cancer cells express low levels of tumor antigens, rendering it difficult for the immune system to recognize and attack these cells^[45]^. The highly heterogeneous nature of breast cancer also leads to some tumor cells being naturally insensitive to ICIs.

Acquired resistance develops progressively during treatment. Mechanisms involve the evolution of immune escape strategies, where tumor cells may reduce antigen expression or upregulate other immunosuppressive molecules through genetic mutations or epigenetic alterations. The immunosuppressive microenvironment may become more pronounced during treatment, evidenced by an increase in Tregs and cancer-associated fibroblasts^[46]^. Tumor cells may also activate alternative survival signaling pathways, such as the PI3K-AKT-mTOR and MAPK pathways, to counteract therapeutic stress^[47,48]^. Additionally, treatment may induce remodeling of the TME, promoting tumor growth and immune escape^[49]^.

## CURRENT APPLICATION OF ICIS IN THE TREATMENT OF BREAST CANCER

Immune checkpoints are a class of immunosuppressive molecules expressed on immune cells that regulate the degree of immune activation. The immune system modulates the body’s response to pathogens by activating inhibitory checkpoint pathways, preventing an overactive immune response that could target normal cells, leading to tissue damage and reduced autoimmune activity^[50]^. However, certain cancer cells use these inhibitory checkpoints to avoid recognition and attack by the immune system^[51]^, such as PD-L1 and CTLA-4, which bind to the checkpoint PD-1 on T cells, thereby blunting the activity of T cells and inhibiting their attack on cancer cells. Tumor cells protect their activity and growth by creating an immunosuppressive microenvironment that allows immune escape. Based on this immune escape mechanism, a series of ICIs have been developed^[52]^, which can bypass the tumor’s immunosuppressive mechanisms and activate cell-mediated antitumor responses. Research on ICIs has revolutionized systemic antitumor therapy for various malignancies, including breast cancer^[44]^.

### TNBC

TNBC is defined by the absence of estrogen and progesterone receptors and HER2^[53]^. It is highly invasive and lacks specific targets and targeted therapies^[54]^. TNBC has a distinct TME characterized by higher PD-L1 expression on tumors and immune cells and higher tumor-infiltrating lymphocyte (TIL) density^[49,54]^.

Immunotherapy is a promising strategy for the treatment of TNBC, with ICIs targeting immunosuppressive receptors such as CTLA-4 and PD-1 to enhance the cytotoxicity and proliferation of TILs^[18,55]^. Previous studies have concluded that PD-L1 inhibitors are particularly effective in certain types of breast cancer, especially in patients with PD-L1 expression^[56]^. In 2020, the US Food and Drug Administration (FDA) approved the PD-L1 inhibitor Atezolizumab in combination with the chemotherapeutic drug paclitaxel for the treatment of PD-L1-positive locally advanced or metastatic TNBC^[57]^.

The early KEYNOTE-012 clinical study of Pembrolizumab in 32 pretreated and primed PD-L1-positive TNBC patients showed an objective response rate of 18.5%^[58]^, suggesting that ICIs may have therapeutic potential in specific subgroups. However, as the study progressed further, the larger KEYNOTE-086 clinical study^[25]^, which enrolled 170 patients with PD-L1 unselected pretreated tumors, showed an overall survival of only 5.3%, significantly lower than the earlier study. In addition, other PD-L1 inhibitors, such as avelumab and atezolizumab, have been explored in the TNBC field^[59]^. In the phase Ib JAVELIN trial^[26]^, avelumab had an objective response rate of 5.2% in 58 heavily pretreated patients. Similarly, Atezolizumab had an objective response rate of 10% in 116 pretreated patients in its phase I trial, but no response was observed in the PD-L1-negative subset^[60]^.

The above findings suggest that although ICIs have shown efficacy in some patients, their effectiveness as monotherapy is relatively limited in the broader TNBC patient population, particularly in the absence of PD-L1 screening.

### Hormone receptor-positive, HER2-negative breast cancer

Hormone receptor-positive, HER2-negative (ER^+^/HER2^-^) breast cancers are characterized by insufficient T cell infiltration, weaker immune responses, lower PD-L1 expression, lower levels of TILs, genomic instability, and lower TMBs, and are also a major contributor to poor therapeutic outcomes when ICIs are used alone^[61]^. A total of 25 ER-positive, HER2-negative advanced PD-L1-positive breast cancer patients were treated with Pembrolizumab monotherapy in the previous KEYNOTE-028 clinical trial, which demonstrated an objective response rate of 12% and a clinical benefit rate of 20%^[27]^. Another Phase I JAVELIN clinical trial study evaluating avelumab monotherapy in patients with all metastatic breast cancer (MBC) subtypes included 72 patients with ER^+^/HER2^-^ breast cancer and showed an objective response rate of only 2.8% and a response independent of tumor PD-L1 expression^[26]^.

Therefore, to enhance the therapeutic effect of ICIs, we need to combine them with other therapies that can promote the body’s antigenic immune response, thereby strengthening the immune system’s ability to recognize and attack tumors. Thermal ablation or other local therapeutic techniques not only directly destroy tumor cells but also promote the release of tumor-associated antigens (TAAs), thereby activating the body’s immune response. This combination therapy strategy further enhances the immune-mediated antitumor activity of ICIs. In patients with less immunogenic ER^+^/HER2^-^ breast cancer, this combination therapy may significantly improve patient prognosis.

### HER2-positive breast cancer

Anti-HER2-positive breast cancers are generally considered to be more immunogenic than ER^+^, HER2-negative tumors, but less so than TNBC^[61]^. There are also variations in immunogenicity across different intrinsic molecular subtypes. HER2-rich subtypes exhibit elevated levels of TILs and increased expression of immune-activating genes. Moreover, certain HER2-targeted therapies are immunogenic, capable of activating antibody-dependent cell-mediated cytotoxicity.

The PANACEAIb/Phase II clinical trial (NCT02129556) evaluated the efficacy of the combination of pembrolizumab and trastuzumab in trastuzumab-resistant HER2-positive MBC patients, with a total enrollment of 58 patients. The study results showed an objective response rate of 15% in PD-L1-positive patients^[62]^. Another Phase II KATE2 clinical trial evaluated the efficacy of the combination of Atezolizumab and trastuzumab emtansine in patients with HER2-positive MBC and found that the 1-year overall survival (OS) rate was higher in the PD-L1-positive group than in the PD-L1-negative group^[63]^.

The combination of ICIs with HER2-targeted agents is considered a new hope for HER2^+^ breast cancer patients who are insensitive to trastuzumab treatment. However, current clinical studies of pembrolizumab or atezolizumab in combination with HER2-targeted agents in patients with resistant HER2-positive breast cancer reveal limited clinical therapeutic efficacy of ICIs. This may be related to the weak immunogenicity and low content of tumor-infiltrating T cells in HER2-positive breast cancer. Therefore, it is necessary to continue exploring therapeutic options that can enhance the body’s immune response to tumor antigens, in order to synergize with ICIs and achieve better therapeutic effects.

### Risks and adverse reactions

ICIs have demonstrated significant progress in treating various cancers in recent years. However, their application in breast cancer treatment still encounters a series of complex adverse reactions and potential risks. ICIs activate the immune response by lifting the inhibition of T cell activity, and although they have shown efficacy in tumor suppression, they are associated with a significant increase in immune-related adverse events (irAEs)^[64,65]^. Common irAEs include dermatologic reactions, gastrointestinal symptoms, hepatotoxicity, and endocrine disturbances. Additionally, although rare, the occurrence of cardiotoxicity, such as myocarditis and pericarditis, remains a concern^[66]^. These reactions not only impact the patient’s quality of life but can also be life-threatening, especially if not promptly recognized and managed. Treatment with ICIs may also elevate the risk of infection, particularly when combined with other immunosuppressive therapies^[67]^. Moreover, some patients may experience allergic reactions ranging from mild injection site reactions to severe anaphylaxis^[68]^. Other common side effects, including fatigue, fever, decreased appetite, and arthralgia, also require attention in clinical management. It is important to note that the long-term risks of ICIs in breast cancer treatment remain unclear; therefore, during ICI monotherapy for breast cancer, patients should be closely monitored, any discomfort should be promptly reported, and the treatment regimen adjusted according to the individual response.

## CLINICAL TRIALS AND RESEARCH ON RESISTANCE TO ICIS

Immunotherapy for breast cancer, particularly ICIs like anti-PD-1 and anti-PD-L1 antibodies, has achieved significant clinical progress by activating the immune system to attack tumors. However, resistance to ICIs is widespread, primarily due to the immunosuppressive state of the TME and tumor heterogeneity. To overcome this resistance, current clinical trials and studies focus on combining other therapeutic approaches to enhance efficacy and overcome resistance. These combined treatment strategies include chemotherapy, targeted therapy, radiotherapy, and modulation of the TME.

### Chemotherapy combined with ICIs

Chemotherapy enhances the immune system’s ability to recognize and attack tumors by killing tumor cells, releasing tumor antigens, and inducing immunogenic cell death (ICD) through chemostimulation. The KEYNOTE-355 study evaluated the efficacy of pembrolizumab in combination with chemotherapy in patients with TNBC and showed that in patients with a combined positive score (CPS) ≥ 10, the median progression-free survival (PFS) was 9.7 months for pembrolizumab combined with chemotherapy compared to 5.6 months for placebo combined with chemotherapy, significantly improving patient PFS^[69]^. In addition, some chemotherapeutic agents, such as cyclophosphamide and adriamycin, further enhance the antitumor effects of ICIs by modulating other components of the immune system, such as enhancing T cell function and suppressing Tregs^[70]^. However, according to the KEYNOTE-522 and IMpassion03 trials, approximately 40% of TNBC patients still fail to achieve pathologic complete remission (pCR), highlighting the need to explore more effective combination therapy strategies^[71]^.

### Targeted therapy combined with ICIs

The combination of targeted therapeutic agents, such as HER2 inhibitors and PI3K inhibitors, with ICIs can enhance the immune response through multiple mechanisms. The IMpassion130 study evaluated the efficacy of atezolizumab in combination with nab-paclitaxel in patients with PD-L1-positive TNBC. The results showed that among PD-L1-positive patients, the median OS was 25.4 months in the atezolizumab plus nab-paclitaxel group compared to 17.9 months in the placebo plus nab-paclitaxel group. For PD-L1 IC-negative patients, the median OS was 19.7 months in both treatment groups, confirming the efficacy of this combination therapy in PD-L1-positive TNBC patients^[72]^. Targeted therapy not only halts the growth of tumor cells by inhibiting specific molecular targets but also enhances the immune system’s ability to attack tumor cells by modifying the TME. Additionally, PI3K inhibitors can enhance immune cell activity by inhibiting tumor cell metabolic pathways, thereby reducing tumor cell nutrient demand^[73]^. Targeted therapeutic agents can also improve the efficacy of ICIs by affecting the antigen-presenting mechanisms of tumor cells and increasing antigen expression on their surface^[74]^.

### Radiotherapy combined with ICIs

Radiation therapy for breast cancer has become a standard treatment option in clinical practice, particularly after breast-conserving surgery for early-stage breast cancer and in the treatment of certain locally advanced cases. For patients with locally recurrent or MBC, radiation therapy can be employed to control lesions, alleviate symptoms, and extend survival to some extent. Furthermore, preclinical and early clinical data suggest that prior radiotherapy enhances antigen release by inducing apoptosis and ICD in tumor cells, and that synergy with modern immunotherapies amplifies the effects of ICIs^[75,76]^. According to a phase II clinical study, pembrolizumab in combination with radiotherapy resulted in an overall remission rate (ORR) of 17.6% in 17 patients with metastatic TNBC, which was higher than that observed in patients previously treated with ICIs as monotherapy^[77]^. Demaria *et al.* in 2005 explored the effects of combined radiotherapy and ICIs on mouse models of breast cancer, particularly in a poorly immunogenic mouse model of breast cancer, where the combination of local radiotherapy and anti-CTLA-4 immune checkpoint inhibition significantly prolonged survival and reduced lung metastasis. Follow-up studies further demonstrated that fractionated radiotherapy in combination with anti-CTLA-4 treatment induced a more significant systemic antitumor effect in a mouse breast cancer model compared with single-dose radiotherapy^[78,79]^. These findings provide clinical rationale for the combination of radiotherapy and ICIs in the treatment of breast cancer.

### Limitation

Although the combination of ICIs with chemotherapy, targeted therapy, and radiotherapy has shown clinical potential in breast cancer treatment, significant limitations persist. A primary challenge of combining ICIs with chemotherapy lies in their potential interactions. Chemotherapy reduces tumor burden by nonspecifically targeting rapidly dividing cells, yet it may also impair the patient’s immune system, diminishing the immune-activating effects of ICIs^[80]^. Targeted therapies can precisely attack specific tumor driver genes or proteins; however, tumor cells often develop resistance through genetic mutations or other adaptive mechanisms. This resistance not only reduces the efficacy of targeted therapies but may also diminish the overall effectiveness of combination therapy with ICIs^[37]^. The limitations of combining radiotherapy with ICIs primarily involve balancing the local effects of radiotherapy with the systemic immune activation induced by ICIs. While radiotherapy can enhance the effects of ICIs by increasing tumor antigen release, it may also damage normal tissues, potentially inducing a widespread immunosuppressive response that weakens the therapeutic efficacy of ICIs^[81]^. Furthermore, chemotherapy, targeted therapy, and radiotherapy may all increase the incidence of irAEs, thereby affecting overall patient tolerability. Thus, novel and more effective combination therapy strategies must be explored.

## TLA

In recent years, TLA has been widely used in the treatment of primary tumor lesions and their metastatic foci, becoming an important part of comprehensive tumor therapy strategies^[82]^. This technique is mainly guided by imaging equipment. The ablation needle is percutaneously penetrated into the tumor tissue, directly leading to the necrosis of tumor cells through physical or chemical methods, causing the tumor to be inactivated and gradually absorbed by the body to become smaller. The principles include thermal and non-thermal effects. Thermal ablation techniques mainly include high-temperature ablation [such as RF ablation and microwave ablation (MWA)] and low-temperature ablation [such as cryoablation (Cryo)]^[83,84]^, while non-thermal ablation mainly refers to irreversible electroporation (IRE)^[85]^. TLA technology not only has the advantages of less trauma and faster recovery but also activates the body’s antitumor immune response, further enhancing the effectiveness of ablation therapy.

### Radiofrequency ablation

Radiofrequency ablation (RFA) is a procedure in which an electric current is applied to conductive ions in the tissue surrounding the electrodes, triggering high-frequency vibrations and generating temperatures of 60-100 °C. This high temperature gradually spreads to the surrounding tissues through heat conduction, eventually leading to necrosis of the tumor tissue. In a clinical study comparing the efficacy of RFA and surgical excision in the treatment of early-stage breast cancer smaller than 2 cm in diameter, Garcia-Tejedor *et al.* found that RFA was effective in controlling tumor progression and was more minimally invasive compared to surgical excision^[86]^. In this process, RFA not only kills tumor cells but also leads to a decrease in the number of Tregs and the release of TAAs, which attenuates immunosuppression, promotes the maturation of DCs, and activates T cell-mediated antitumor immune effects. In addition, RFA induces an increase in the levels of pro-inflammatory cytokines and tumor necrosis factor-α (TNF-α), which further enhances the antitumor immune effects of the body^[87-89]^.

Although RFA has demonstrated potential in enhancing the body’s antitumor immunity, as a monotherapy, it has a high rate of disease progression and local recurrence of tumors^[90]^. The main reason for this is that the induced immune response is mostly limited to the ablation site and unable to extend effectively throughout the body, and the immune activation is neither sufficiently comprehensive nor long-lasting^[88]^. In addition, although RFA reduces the number of Treg cells, other mechanisms of immunosuppression may persist. Therefore, RFA often needs to be combined with other treatments to enhance efficacy.

### MWA

MWA is a technique that generates electromagnetic waves at a frequency of 900-2,450 MHz, causing water molecules in tissues to resonate and generate heat^[89,91]^. This method is particularly effective for tissues with high water content, as the high-frequency vibration of water molecules rapidly generates intense heat^[92]^. Compared to RFA, MWA has a faster heat-up rate and is relatively unaffected by thermal deposition effects, allowing for a wider heating range^[93]^. While this process helps the immune system to recognize and respond, the antigenic denaturation and coagulative necrosis induced by MWA are more pronounced, resulting in a weaker T cell response that is less favorable to the activation of antitumor immune effects^[94,95]^. More pronounced cellular necrosis diminishes active antigen presentation, thereby decreasing the likelihood of activating an effective immune response. Furthermore, the hyperthermia produced by MWA also inhibits immune cell activity^[96,97]^. Therefore, despite the advantages of rapid and extensive heating, MWA may have limitations in activating antitumor immunity. To improve the antitumor immune activation effect of MWA, future studies could explore combining it with immune-boosting agents such as ICIs, thereby enhancing its overall efficacy in cancer therapy.

### Cryo

Cryo is a therapeutic technique that uses extremely low temperatures to rapidly cause necrosis, apoptosis, and intravascular microthrombosis in tumor cells, leading to the death of the tumor tissue^[98]^. The Cryo technique more commonly used in clinical practice is argon-helium Cryo, which uses argon and helium as a medium to destroy tumor cells by rapidly achieving low temperatures^[99]^. Unlike the coagulative necrosis induced by thermal ablation, the liquefactive necrosis induced by Cryo better preserves the immunogenicity of the antigen (undenatured), which is more favorable for antigen presentation and T cell activation^[98,100]^. A study by Sabel *et al.* found that rapid cooling reduced tumor-draining lymph nodes (TDLN) and prolonged survival time in mice by establishing a mouse breast cancer ablation model^[101]^. This is because Cryo induces tumor-specific T cell responses in the TDLN, increases systemic natural killer cells (NK cells) activity, and reduces Treg cells, thereby attenuating suppression of the immune system^[102]^. In addition, Cryo promotes the secretion of pro-inflammatory factors more than thermal ablation, thereby triggering stronger antitumor immune effects^[99,103]^.

Cryo therapy has demonstrated a certain degree of effectiveness in destroying tumor cells, but it is deficient in uniformly covering the tumor area, preventing tumor recurrence, and avoiding damage to the surrounding normal tissues. Meanwhile, the formation of microthrombi after Cryo hinders the infiltration of immune cells to a certain extent^[104,105]^. The antitumor immune effect triggered by Cryo may not be sufficient to eradicate all tumor cells, and there is a wide variation in efficacy between individuals. These factors make Cryo therapy challenging and limited in achieving immune effects, and further studies are needed to optimize its efficacy.

### IRE

IRE is an emerging oncology technology in recent years. Unlike traditional thermal and Cryo, IRE does not rely on high or low temperatures to destroy tumor cells but rather applies high-voltage electrical impulses to form tiny nanoscale pores in the tumor cell membranes, altering the permeability of the cell membranes. This alteration disrupts the osmotic balance between the cell’s internal and external environments, ultimately leading to apoptosis of the tumor cells^[106,107]^.

Shao *et al.* simulated the effects of thermal ablation (50 °C for 30 min), Cryo (-80 °C for 30 min), and IRE (1,250 V/cm, 50 μs, 99 pulses, 1 Hz) on the antigen release and T cell activation of malignant melanoma cells using an *in vitro* model^[108]^. The results showed that IRE released more antigenic and immunogenic activity compared to thermal and Cryo, thus triggering a stronger antitumor immune effect. Pastori *et al.* compared the therapeutic effects of IRE with RFA in a mouse model of breast cancer and found that IRE not only increased the release of ICD markers but also decreased the release of cytokines associated with tumor recovery and metastasis^[109]^. Therefore, IRE has a significant advantage in triggering antitumor immune responses.

However, IRE therapy still has some limitations in triggering antitumor effects. Firstly, the non-homogeneous electrical properties of the tumor tissue lead to inhomogeneous distribution of the high-voltage pulsed electric field, resulting in incomplete ablation and increasing the risk of tumor recurrence^[110,111]^. In addition, in clinical application, the treatment effect of IRE may be affected by factors such as tumor location, size, and surrounding tissue structure due to the high requirements of IRE on the arrangement of electrode needles around the lesion^[112]^. Nonetheless, IRE demonstrates significant potential to enhance immune responses and may be used in combination with ICIs in the future to improve overall antitumor effects.

## TLA COMBINED WITH ICIS IN THE TREATMENT OF BREAST CANCER

Although ICIs have shown significant efficacy in treating various cancers, some cancers still exhibit tolerance or resistance to these therapies^[113-115]^. Breast cancer, especially TNBC, is a common cancer type resistant to ICIs^[116-118]^. This tolerance may be caused by various mechanisms, including high immunosuppression in the TME^[119-121]^, lower TMB^[122]^, and lower neoantigen (Neo) expression^[123,124]^. These mechanisms allow cancer to evade the immune system by modulating the number of immunosuppressive cells, downregulating the expression of immune-related molecules, and altering T cell function^[125,126]^. TLA overcomes the resistance mechanisms of ICIs by enhancing the delivery and infiltration of specific T cells into the tumor site, thereby improving ICIs’ efficacy. Clinical and animal trials combining TLA with ICIs in breast cancer treatment have yielded promising results [Tables 1 and 2]. The mechanisms of TLA combined with ICIs for overcoming drug resistance in breast cancer include the following aspects.

**Table 1:** Clinical trial of TLA combined with ICIs in the treatment of breast cancer

**Identifer**	**Title**	**Phase**	**Study design**	**Type of tumor**	**Type of ICIs**	**Type of TLA**	**Primary outcome measure**	**Estimated study completion date**
NCT 06246968	A Study of Pembrolizumab and Cryoablation in People With Breast Cancer	Phase 1	Randomized	Metastatic TNBC	Pembrolizumab	Cryo	Change in CD4-PD1 from baseline to post-Cryo	January 29th, 2027
NCT 04249167	Cryoablation, Atezolizumab/Nab-paclitaxel for Locally Advanced or Metastatic Triple Negative Breast Cancer	Early phase 1	Single group assignment	Locally advanced or metastatic PD-L1 positive TNBC	Atezolizumab	Cryo	Safety and feasibility of Cryo with systemic atezolizumab/nab-paclitaxel	November 17th, 2021
NCT04805736	Microwave Ablation Combined With Camrelizumab in the Treatment of Early Breast Cancer	Phase 2	Parallel assignment	Early-stage breast cancer	Camrelizumab	MWA	Safety of MWA combined with camrelizumab	April 30th, 2023
NCT02833233	A Study of Preoperative Treatment With Cryoablation and Immune Therapy in Early Stage Breast Cancer	Not applicable	Single group assignment	Early stage breast cancer	Ipilimumab/nivolumab	Cryo	Number of adverse events	June 7th, 2024

TLA: Tumor local ablation; ICIs: immune checkpoint inhibitors; TNBC: triple-negative breast cancer; PD-L1: programmed death ligand-1; Cryo: cryoablation; MWA: microwave ablation.

**Table 2:** Experimental study of TLA combined with ICIs in the treatment of breast cancer

**Study**	**Year**	**Objective**	**Type of tumor**	**Type of ICIs**	**Type of TLA**	**Therapeutic effect**
Enhanced antitumor efficacy through microwave ablation in combination with immune checkpoints blockade in breast cancer: A preclinical study in a murine model	2018	Mouse	4T1 subcutaneous tumor	AntiPD-1/anti-CTLA-4	MWA	synergistically enhance antitumor efficacy with augmented specific immune responses
Intratumoral Plasmid IL12 Expands CD8+ T Cells and Induces a CXCR3 Gene Signature in Triple-negative Breast Tumors that Sensitizes Patients to Anti-PD-1 Therapy	2021	Mouse	Mouse models of TNBC	AntiPD-1	Tavo	Intratumoral administration of Tavo significantly suppressed tumor growth and improved survival

TLA: Tumor local ablation; ICIs: immune checkpoint inhibitors; PD-1: programmed cell death protein 1; CTLA-4: cytotoxic T-lymphocyte-associated antigen 4; MWA: microwave ablation; TNBC: triple-negative breast cancer; Tavo: tavokinogene telseplasmid.

### Enhanced antigen release and presentation

TLA in breast cancer treatment is not only limited to direct physical destruction but also accompanied by a complex set of biological responses that, in turn, affect the tumor’s response to ICIs.

Once the tumor cells are destroyed during ablation, the damaged cells release a large number of TAAs and damage-associated molecular patterns (DAMPs), triggering an immune response^[127]^. These molecular patterns are recognized by the immune system, especially by antigen-presenting cells such as DCs, which capture and present them to T cells, thereby initiating and enhancing the body’s immune response^[128]^. TAAs and DAMPs not only activate the intrinsic immune system but also promote an adaptive immune response that establishes the basis for further clearance of breast cancer^[129]^. Guo *et al.* found that a single IRE treatment led to complete regression of poorly immunogenic metastatic 4T1-Luc mouse mammary carcinomas and that IRE-treated 4T1 cells showed the release of DAMPs, including calreticulin, HMGB1, and ATP, and activation of DCs^[130]^. This shows that IRE not only eliminates local tumors but also triggers an antitumor immune response. At the same time, the release of heat shock proteins (HSPs) and other inflammatory mediators further enhances the recognition and attack of tumors by the immune system^[127]^. HSPs act as molecular chaperones, helping DAMPs and TAAs to be delivered and displayed to promote an immune response. This immune response is not limited to the ablation site but is systemic, meaning that other potential metastatic tumor cells can also be recognized and attacked. This process significantly enhances the antitumor capacity of the body’s immune system.

ICIs play a crucial supporting role in this process. Typically, tumor cells help to evade immune surveillance by expressing proteins such as PD-L1, which interact with the PD-1/PD-L1 pathway on T cells, thereby inhibiting the killing function of T cells^[16]^. ICIs keep T cells active and capable of killing by blocking these inhibitory signals^[131]^. Regen-Tuero *et al.* demonstrated that Cryo not only enhances the immunogenicity of tumors and boosts the therapeutic efficacy of ICIs but also relieves immune suppression through immune checkpoint inhibition, leading to a robust immune response to the tumor-specific antigens released by Cryo^[132]^.

Through this process, TLA effectively addresses the issue of tumor drug resistance. First, TLA reduces the number of drug-resistant cells by physically destroying tumor cells. Secondly, the immune response triggered by TLA can recognize and attack the residual drug-resistant tumor cells, and ICIs alleviate immunosuppression, thereby enhancing the potency and durability of T cells.

### Inhibition of angiogenesis and increased immune infiltration

TLA demonstrates considerable therapeutic potential in inhibiting tumor angiogenesis and enhancing immune infiltration, providing a crucial basis for its combination with ICIs. First, TLA directly disrupts the vascular network within the tumor through physical means, resulting in a significant reduction in blood supply and thereby inhibiting tumor growth and expansion. Moreover, TLA modifies the TME by inhibiting the angiogenic process, as evidenced by decreased secretion of vascular endothelial growth factor (VEGF) and other pro-angiogenic factors. This effect not only reduces neovascularization, thereby limiting nutrient supply to the tumor, but also promotes tumor cell apoptosis^[133]^. Secondly, TLA may indirectly enhance the TME and reduce its immunosuppressive properties, making it more conducive to the infiltration and activation of effector immune cells^[133,134]^. Specifically, TLA reduces the abundance of immunosuppressive cells while enhancing the recruitment and activation of effector immune cells. These effector immune cells are more likely to infiltrate the tumor site following ablation and effectively eliminate residual tumor cells by releasing cytotoxic molecules^[135]^. This remodeling of the local environment not only directly diminishes tumor viability but also facilitates the establishment of antitumor immune memory by enhancing adaptive immune responses. The enhancement of the immune environment triggered by TLA creates favorable conditions for the action of ICIs. By blocking immune checkpoint pathways such as PD-1/PD-L1 or CTLA-4, ICIs lift the functional inhibition of T cells, thereby enabling solid tumors, including breast cancer, which are initially insensitive to ICIs, to exhibit stronger immune responses in combination therapy.

### Reducing the inhibitory properties of the TME

The TME plays a critical role in cancer development and progression and usually has immunosuppressive properties, thus inhibiting the activity of immune cells and helping tumor cells evade the body’s immune surveillance and attacks^[136]^. Immunosuppressive cells, such as Tregs and MDSCs, normally play a protective role in tumor growth within the TME^[137]^. TLA treatment effectively reduces the number of these suppressor cells, thereby releasing immune effector cells from suppression. In addition, ablation can increase the infiltration of immune effector cells, such as cytotoxic T lymphocytes (CTLs) and NK cells^[131]^. Furthermore, TLA also helps to alter the cytokine profile in the TME. Normally, pro-inflammatory and anti-inflammatory cytokines in the TME are in a complex balance. Ablative therapy can disrupt this balance by promoting the release of inflammatory cytokines such as IL-1β, IL-6, and TNF-α, enhancing the local immune response, and reducing the levels of anti-inflammatory cytokines^[103,138]^. This release of inflammatory mediators creates a pro-inflammatory microenvironment conducive to an immune system response, thereby enhancing the killing and clearance of residual tumor cells. This has been confirmed by previous studies. Lou *et al.* used cryo-thermal therapy to treat mouse mammary cancer, demonstrating that it induced Th1-dominant differentiation and specifically targeted Treg fragility by downregulating TNF-α levels, thus achieving a long-term antitumor immune response^[139]^.

The immunosuppressive properties of the TME can be significantly reduced by combining TLA with ICIs. TLA induces a local inflammatory response and improves the infiltration environment of immune cells by physically destroying the tumor and its surrounding immunosuppressive cells. Meanwhile, ICIs enhance the antitumor immune response by blocking the inhibitory signaling pathway between tumor cells and immune cells, and by alleviating the T cell inhibitory signal. Together, they enable the immune system to more effectively recognize and attack tumor cells, thereby addressing drug resistance and enhancing the effectiveness of breast cancer treatment.

### Promote memory immune response

TLA primarily targets visible tumor lesions, but some tiny or hidden metastatic lesions may not be completely eliminated. This is because, while TLA is effective in destroying known tumor tissue, it is difficult to detect and deal with metastatic tumor cells that are not yet visible or are hidden in location^[140]^. However, the combination of TLA and ICIs can effectively address these challenges by promoting memory immune responses^[141]^. In a study by Li *et al.* on MWA combined with OK-432 for the treatment of a mouse model of breast cancer, it was found that MWA was not only able to activate the T cell immune response in breast cancer, but in combination with OK-432, was also able to induce an organismal Th1-type response and trigger specific antitumor immunity^[142]^. In addition, the combination of TLA with ICIs, such as anti-PD-1 antibodies, was also able to prolong the survival time of patients^[130,143]^.

ICIs reduce the risk of recurrence and metastasis by blocking inhibitory signals on T cells, thereby enhancing the systemic immune response and aiding in the clearance of small, undetected metastatic foci. A study by Zhu *et al.* found that combining MWA with PD-1 and CTLA-4 blockers to treat a 4T1 hormonal mouse model significantly prolonged the survival time of the mice and protected the majority of surviving mice from relapse^[94]^. By disarming inhibitory signals on immune cells, these inhibitors enable T cells to more widely and effectively recognize and attack tumor cells scattered throughout the body, including those tiny metastatic foci that have not yet been detected. In this way, ICIs are able to compensate for the shortcomings of local ablation and thus achieve a more comprehensive antitumor effect.

Meanwhile, an effective immune response not only relies on the immediate activity of effector T cells but also requires the establishment of long-term immune memory. Durable antitumor immune memory is essential to prevent long-term tumor recurrence. The therapeutic modality of TLA combined with ICIs promotes the formation of specific antitumor immune memory *in vivo*. Babikr *et al.* used IRE combined with a Toll-like receptor (TLR) 3/9 agonist (poly I/CpG) and a PD-1 blocker for the treatment of primary and distant EG7 tumors and found not only complete eradication of the primary tumor but also complete elimination of the concomitant ~100 mm^3^ EG7 tumor and BL6-100OVA lung metastases. Furthermore, IRE combined with combination therapy showed significant therapeutic efficacy in two mouse breast cancer models (Tg1-1 and 4T1)^[144]^. Through this combined treatment model, a strong and durable antitumor immune memory can be built up in the body, thus providing an important safeguard against long-term tumor recurrence. This persistent immune memory not only fights residual tumor cells in the short term but also monitors and removes emerging tumor cells in the long term, significantly improving the long-term effectiveness of treatment and patient survival.

Through the combined treatment approach of TLA and ICIs, a robust and enduring antitumor immune memory can be established in the body, effectively addressing drug resistance in tumors and preventing long-term recurrence. This enduring immune memory plays a pivotal role in overcoming drug resistance. Initially, TLA physically destroys tumor cells and ICIs alleviate immunosuppressive signals, enhancing the immediate killing efficacy of T cells against residual tumor cells. Furthermore, in the long term, ICIs boost the development and functionality of memory T cells, enabling continual surveillance and swift elimination of emerging tumor cells to thwart drug-resistant tumor formation and dissemination, ensuring complete tumor eradication and circumventing the constraints of singular treatment modalities.

## DISCUSSION AND PROSPECT

Despite the promise shown by ablation combined with immunotherapy in basic research and clinical trials, this innovative treatment still encounters numerous challenges. The primary concern is the development of drug resistance. Due to the widespread use of immunotherapy, some breast cancer patients may gradually develop resistance to these therapies, leading to diminished or ineffective treatment outcomes. Therefore, a thorough investigation and understanding of tumor drug resistance mechanisms, particularly in the context of combined ablation and immunotherapy, are crucial for optimizing treatment.

To address these challenges, future research directions should focus on the following areas: First, advanced technologies such as genomics, proteomics, and single-cell sequencing should be employed to thoroughly investigate the mechanisms of drug resistance in breast cancer cells when treated with a combination of TLA and ICIs. The focus should be placed on identifying the key genes and signaling pathways driving drug resistance, particularly those potentially activated by TLA-induced changes in the TME. These findings will aid in the development of novel ICIs or adjuvant drugs aimed at enhancing therapeutic efficacy and delaying the onset of resistance by targeting these pathways.

Second, treatment strategies should be further refined to identify the optimal combination of TLA and ICIs. Specifically, studies should assess the various biological effects of different types of ablative therapies combined with ICIs and their relevance to different breast cancer subtypes. Optimizing treatment timing, dosage, and frequency is crucial for maximizing the benefits of combination therapies. Additionally, clinical trials should investigate the effects of combining TLA with ICIs at various treatment stages to identify the most effective and safe treatment modalities. However, managing irAEs in combination therapy with TLA and ICIs presents a significant challenge. These adverse effects may include skin toxicity, gastrointestinal reactions, endocrine disruption, and more severe cardiac or pulmonary complications. Effective management strategies involve close monitoring of patients’ immune status, early identification and intervention for irAEs, and the development of individualized response protocols. Furthermore, research should focus on developing and validating predictive biomarkers to identify patients who are likely to benefit from this combination therapy and those at higher risk for irAEs, thereby guiding treatment decisions more precisely.

Third, the development of personalized treatment strategies is essential. By integrating patients’ genomic information, tumor microenvironmental characteristics, and immune status, treatment targeting and efficacy can be enhanced through biomarker screening and precision medicine. Moreover, research should focus on identifying and validating predictive biomarkers that not only help select patients suitable for combination therapy with TLA and ICIs but also predict the development of therapeutic resistance, thereby guiding early intervention and adjustment of treatment regimens to minimize side effects and enhance patient survival. By conducting in-depth analyses of drug resistance mechanisms, optimizing therapeutic strategies, and developing personalized therapeutic regimens, future research aims to offer more effective and durable treatment options for clinical application.

## CONCLUSION

Current understanding of the mechanisms of drug resistance in breast cancer reveals that tumor cells develop resistance through multiple pathways, such as downregulating antigen expression, altering the TME, and activating alternative immunosuppressive pathways. The complexity and diversity of these mechanisms have significant implications for the development of therapeutic strategies. Specifically, in response to ICIs resistance, tumor cells can evade immune surveillance by upregulating PD-L1 expression, modulating the number and function of immunosuppressive cells, or activating alternative immune checkpoint molecules. To address drug resistance, future therapeutic strategies should focus on multi-target combination therapy, optimizing treatment regimens, and developing personalized therapies. These research directions aim to enhance the efficacy of TLA in combination with ICIs and provide more effective and personalized treatment options for breast cancer patients.

## DECLARATIONS

### Copyright

© The Author(s) 2024.
